# P-1323. Ceftazidime–avibactam in real-world clinical practice: results from a single center

**DOI:** 10.1093/ofid/ofaf695.1511

**Published:** 2026-01-11

**Authors:** Jon Salmanton-Garcia, Carla Niveyro, Victoria Isabel martin, Pedro A Villalba apestegui, lucila maria, maria cynthia tomasino, Ricardo de Jesus Solari Maidana, maria lorena lopez, Claudia Viviana Villalba, Gustavo-Adolfo Méndez

**Affiliations:** University Hospital Cologne, Cologne, Germany, Cologne, Nordrhein-Westfalen, Germany; Madariaga Hospital, Posadas, Misiones, Argentina; Infectious Diseases Department, Hospital Escuela de Agudos Dr. Ramón Madariaga. Posadas, Misiones – Argentina, posadas, Misiones, Argentina; Infectious Diseases Department, Hospital Escuela de Agudos Dr. Ramón Madariaga. Posadas, Misiones – Argentina, posadas, Misiones, Argentina; Infectious Diseases Department, Hospital Escuela de Agudos Dr. Ramón Madariaga. Posadas, Misiones – Argentina, posadas, Misiones, Argentina; Infectious Diseases Department, Hospital Escuela de Agudos Dr. Ramón Madariaga. Posadas, Misiones – Argentina, posadas, Misiones, Argentina; Infectious Diseases Department, Hospital Escuela de Agudos Dr. Ramón Madariaga. Posadas, Misiones – Argentina, posadas, Misiones, Argentina; Infectious Diseases Department, Hospital Escuela de Agudos Dr. Ramón Madariaga. Posadas, Misiones – Argentina, posadas, Misiones, Argentina; Infectious Diseases Department, Hospital Escuela de Agudos Dr. Ramón Madariaga. Posadas, Misiones – Argentina, posadas, Misiones, Argentina; Hospital Escuela de Agudos Dr Ramón Madariaga, Posadas, Misiones, Argentina, Posadas, Misiones, Argentina

## Abstract

**Background:**

Over the past decade, rising multidrug resistance (MDR) in Gram-negative bacteria has become a global health emergency and clinical challenge. This study examines clinical features, resistance patterns, antibiotic therapy, and mortality outcomes.

**Methods:**

We conducted a retrospective, single-center study (Nov 2019–Sept 2024) on hospitalized patients who received ceftazidime–avibactam (±aztreonam) for ≥72 hours. Data included demographics, comorbidities, prior healthcare exposures, clinical severity scores, microbiology, antibiotic regimens, treatment adjustments, and outcomes.

**Results:**

A total of 109 patients were included (62% male, median age 49 [range 16–88]), with a median hospital stay of 31 days (range 4–248). Severe clinical scores were noted in 19% (PITT ≥4), 32% (CCI ≥4), and 38% (INCREMENT ≥8). Central venous catheters were used in 75%, prior antibiotic use in 66%, prior hospitalization in 58%, and urinary catheters in 49%. Hematologic malignancies were present in 35%, mainly acute myeloid leukemia (17%). *Klebsiella pneumoniae* was the predominant pathogen (66%), often isolated alone. Multidrug resistance was common (MBL in 22%, MBL+ESBL in 37%). All patients received empiric antibiotics (median 3 days), most frequently ceftazidime-avibactam with aztreonam (39%). Targeted therapy (median 7 days) was used in 77%, commonly the same regimen (48%). Antibiotic regimens were changed in 77% (escalation 57%, de-escalation 17%). Clinical cure was achieved in 74%, but mortality was 33% (16% infection-related). ICU admission (OR 4.1, p=0.034), INCREMENT ≥8 (OR 3.5, p=0.035), and urinary catheter use (OR 4.6, p=0.025) were independently associated with mortality.

**Conclusion:**

This study highlights the importance of early, targeted antibiotics and comprehensive management for multidrug-resistant (MDR) Gram-negative infections. Ceftazidime–avibactam, particularly with aztreonam, is effective against MDR pathogens, including MBL-resistant strains. ICU admission, high INCREMENT scores, and urinary catheter use are linked to higher mortality, stressing the need for vigilant risk assessment and tailored therapy. These findings offer real-world guidance for clinical decisions in this challenging setting.

**Disclosures:**

Jon Salmanton-Garcia, MSc, MPH, PhD, menarini, gilead, astrazeneca, pfizer: Honoraria

